# Advanced CD276-Targeting Dual-Payload Antibody–Drug Conjugates for Cancer Therapy

**DOI:** 10.1158/2767-9764.CRC-26-0059

**Published:** 2026-04-21

**Authors:** Zhuoxin Zhou, Davis Ballard, Jiashuai Zhang, Zhantao Du, Natsorn Watcharadulyarat, Tanvi Varadkar, Srijita Chowdhury, Sathvik R. Kambam, Phoebe Hu, Selena Khanal, Lufang Zhou, Xiaoguang Liu

**Affiliations:** 1 Department of Chemical and Biomolecular Engineering, The Ohio State University, Columbus, Ohio.; 2 Department of Biomedical Engineering, The Ohio State University, Columbus, Ohio.; 3 Department of Surgery, The Ohio State University, Columbus, Ohio.; 4 The James Comprehensive Cancer Center, The Ohio State University, Columbus, Ohio.

## Abstract

Antibody–drug conjugate (ADC) represents an effective therapeutic strategy for cancer, leveraging a cancer-targeting monoclonal antibody (mAb) linked to a potent payload. In this study, we report novel dual-payload ADCs (DualADC), which harness one antibody to simultaneously deliver chemotherapy and immunotherapy for aggressive triple-negative breast cancer (TNBC). Specifically, we developed two-site, that is, cysteine and lysine, co-conjugation technologies to link a chemotherapeutic agent for direct tumor cell killing and a Toll-like receptor dual agonist for tumoral immunity enhancement to our humanized anti-CD276 (B7-H3) mAb. Our antibody binds to TNBC with high affinity and selectivity while exhibiting minimal off-target effects in normal human tissues. Three DualADCs were fully characterized by validating the conjugations and quantifying the drug-to-antibody ratios and drug-to-drug ratio using several analytic tools. *In vitro* evaluations revealed a high cancer cell binding rate via flow cytometry, efficient drug internalization with confocal microscopy, and high anticancer cytotoxicity in three TNBC cell lines. *In vivo* investigations in two TNBC xenograft mouse models demonstrated that DualADC carrying deruxtecan and imidazoquinoline has the best antitumor efficacy, that is, favorable biodistribution in TNBC, high tumor burden reduction, low systemic toxicity, and upregulation of immune pathways. Collectively, this study establishes advanced dual-payload antibody–drug conjugation technologies and identifies a good DualADC candidate for cancer therapy.

**Significance:**

Advanced dual-payload antibody-drug conjugation technologies were established, and a promising DualADC was developed for targeted cancer chemoimmunotherapy.

## Introduction

Cancer remains a leading cause of mortality in the United States, with an estimated two million new cases and 618,120 cancer-related deaths projected for 2025. Breast cancer accounts for approximately 32% of new diagnoses and 14% of deaths (1). Triple-negative breast cancer (TNBC), which lacks expression of the estrogen receptor, progesterone receptor, and human epidermal growth factor receptor 2 (HER2), is the most aggressive subtype, associated with poor prognosis and survival rates (2, 3). Patients with advanced TNBC often experience recurrence following standard treatments, including surgery, radiotherapy, and chemotherapy (4–7). This study employs TNBC as a model to evaluate the designed novel targeted cancer therapy.

Antibody–drug conjugates (ADC) have been developed to selectively deliver potent payloads, such as microtubule inhibitors, DNA-damaging reagents, or topoisomerase I (TOP1) inhibitors, for cancer treatment (8, 9). By 2025, the US FDA has approved 15 ADCs (10–14), targeting HER2, Nectin-4, trophoblastic cell surface antigen 2 (Trop-2), and others. The anti–Trop-2 ADCs, including sacituzumab govitecan and datopotamab deruxtecan (DXd), have been used to treat breast cancer and other malignancies. Despite their notable advantages (15), clinical effectiveness can be undermined by compensatory mechanisms, the emergence of drug resistance (16–18), and inherent cancer heterogeneity (3, 19). For instance, single-payload ADCs can inhibit TNBC tumor growth, but recurrence often occurs after treatment cessation in animal studies, as observed in our previous animal studies (20, 21). These challenges may be addressed by integrating multiple anticancer mechanisms into a single therapy or through combining therapies. Another clinical hurdle of ADC therapy is minimizing the dose-dependent adverse effects from off-target monoclonal antibody (mAb) activity while maintaining high anticancer efficacy. This can be mitigated by developing or screening new mAbs, engineering the existing antibodies to balance receptor binding affinity and cancer specificity, comparing responses to different payloads, optimizing the drug-to-antibody ratio (DAR), and exploring therapeutic combinations.

To overcome the limitations of conventional single-payload ADCs in cancer treatment, we introduced the concept of dual-payload ADCs (DualADC) via co-conjugation. In a previous study, one mAb was employed to codeliver chemotherapy such as monomethyl auristatin F (MMAF) and immunotherapy such as a Toll-like receptor (TLR) agonist for the first time (21). Our reported design offered significant advantages compared with the DualADCs established by Tsuchikama Lab (22) and Kayser Lab (23), where two cytotoxic microtubules [i.e., prelinked monomethyl auristatin (MMAE) and MMAF (22) or MMAE and DM1 (23)] were conjugated with the well-known anti-HER2 trastuzumab. Although the established mAb-MMAF/imidazoquinoline (IMQ) conjugate reduced tumor burden at a high dose, it was not fully effective against the aggressive cancer. Therefore, this study developed two advanced co-conjugation technologies by applying new linkers to incorporate highly potent payloads and immune-booting reagents. Notably, these DualADC conjugation platforms enable wider choices of chemo- and immunotherapies and tuning of DARs for further optimization of the drug-to-drug ratio (DDR). The new DualADC will be fully characterized and evaluated herein.

As reported in the literature (24–29), the transmembrane protein CD276 (B7-H3) is associated with invasion, metastasis, poor prognosis, and immune tolerance in cancers (30–34). Our analysis using The Cancer Genome Atlas Breast Invasive Carcinoma (TCGA-BRCA) and ProteomicsDB in this study reveals elevated CD276 transcript levels and significantly higher protein expression in TNBC compared with normal breast tissues. Previous immunohistochemistry (IHC) analysis of tissue microarrays from more than 100 samples from patients with TNBC showed high levels of CD276 expression in >70% of cases (21). Recent clinical trials of GSK5764227 (HS-20093) and ifinatamab DXd (NCT06203210, NCT04145622; refs. 35, 36) for prostate cancer and small cell lung cancer demonstrate that CD276 is a promising target, and the derived ADC has manageable toxicity. We have developed and humanized a unique anti-CD276 mAb (21), showing minimal systemic toxicity and low plasma clearance. This study revealed that our mAb possesses highly favorable drug delivery parameters, that is, cancer-specific targeting and biodistribution, suitable affinity, and effective drug delivery of multiple combinations. Thus, we utilized this antibody to construct DualADCs for cancer therapy.

This study constructed three DualADCs comprising a humanized CD276 mAb for TNBC targeting, a cytotoxic chemotherapeutic agent (microtubule inhibitor or TOPI inhibitor), and an immune-boosting reagent (TLR 7/8 agonist). Robust protocols were established to co-conjugate chemo- and immunotherapy at cysteine and lysine residues. These DualADCs were fully characterized for cancer binding, targeting specificity, receptor affinity, drug internalization, DARs and DDR, and cytotoxicity. Finally, *in vivo* evaluations validated tumor specificity, high therapeutic efficacy, minimal toxicity, and synergistic anticancer mechanisms. Taken together, this study establishes advanced DualADCs and conjugation procedures for novel targeted cancer therapy.

## Materials and Methods

The animal studies in this project were conducted according to the protocols 2023A00000039 and 2022A00000071 approved by the Institutional Animal Care and Use Committee. This research was approved by the Institutional Biosafety Committee at the Ohio State University.

### Cell lines and cell culture media

The human TNBC cell lines, including MDA-MB-231 (ATCC, cat. #HTB-26, RRID:CVCL_0062), MDA-MB-231-firefly luciferase (FLuc; GenTarget, cat. #SC059-Puro, RRID:CVCL_YZ80), MDA-MB-468 (ATCC, cat. #HTB-132, RRID:CVCL_0419), and MDA-MB-468-FLuc-GFP (GeneCopoeia, cat. #SL027, RRID:CVCL_C8XW), were used. These TNBC lines were cultured in Dulbecco’s Modified Eagle Medium supplemented with 10% fetal bovine serum (FBS, v/v) and 1% penicillin–streptomycin (Pen-Strep, v/v). Mouse TNBC cell lines 4T1 (ATCC, cat. #CRL-2539, RRID:CVCL_0125) and 4T1-FLuc (ATCC, cat. #CRL-2539-LUC2, RRID:CVCL_5I85) were grown in RPMI 1640 medium supplemented with 10% FBS and 1% Pen-Strep. The rat breast cancer cell line MAT-B-III (ATCC, cat. #CRL-1666, RRID:CVCL_3475) was cultured in McCoy’s 5A Medium (ATCC, cat. #30-2007) supplemented with 10% FBS and 1% Pen-Strep. The seed cells of Chinese hamster ovary (CHO) K1 for mAb production were maintained in Dynamis medium in 125-mL shaker flasks at an agitation speed of 130 rpm. All cell cultures were maintained at 37°C with 5% or 8% CO_2_ in a humidified incubator (Eppendorf). All cell culture media and bioreagents were purchased from Fisher Scientific unless otherwise specified. All the cell lines were purchased commercially, authenticated with genetic profiling for polymorphic short tandem repeat analysis at the University Genomics Core, and confirmed with an in-house mycoplasma test using PCR primers that amplify sequences of 16S rRNA genes. The latest test date of all cell banks with stock vials of 30 to 100 was November 21, 2022. The length of time between cell thaw of the tested cell bank and use in our experiment was 2 to 3 weeks.

### mAb production

The humanized anti-CD276 mAb was produced using the CHO K1 production line in Dynamis medium, initially supplemented with 4 mmol/L L-glutamine and 6 g/L glucose. Fed-batch mAb production was performed in 2-L benchtop stirred-tank bioreactors with process parameters of 37°C, agitation at 140 rpm, dissolved oxygen at 40%, and pH 7.0. The bioreactor culture was fed with 10% (v/v) of Efficient Feed C nutrients, 4 mmol/L L-glutamine, and 6 g/L glucose. Spent medium was harvested when culture viability dropped to 50% and stored at −80°C until purification. The mAb was purified using liquid chromatography (Bio-Rad) equipped with a 5-mL UNOsphere SUPrA Protein A column. The raw mAb harvest was loaded with mobile phase A (0.02 mol/L Na_3_PO_4_, 0.02 mol/L Na_3_C_6_H_5_O_7_, pH 7.5) and eluted using mobile phase B (0.1 mol/L NaCl, 0.02 mol/L Na_3_C_6_H_5_O_7_, pH 3.0) at a flow rate of 5 to 10 mL/minute. The purified mAb was buffer-exchanged with phosphate-buffered saline (PBS) and concentrated to 2 or 5 mg/mL using a 10 kDa molecular weight cut-off (MWCO) polyethersulfone (PES) ultrafiltration column. The purified mAb was stored at 4°C for the short term or at −20°C for the long term.

### Affinity assay

An indirect ELISA was performed to evaluate the binding affinity of CD276 mAb to the receptors across species (human, monkey, mouse, and rat). Briefly, 96-well high-binding plates were coated with 100 μL of 2 μg/mL CD276 antigen in 50 mmol/L Na_2_CO_3_-NaHCO_3_ buffer (pH 9.6) overnight at 4°C. Commercial receptors derived from human (Sino Biological, cat. #11864-H08H), monkey (MedChemExpress, cat. #HY-P7639), mouse (MedChemExpress, cat. #HY-P70125), and rat (Sino Biological, cat. #80380-R02H) were used. Plates were washed 3 times with PBS containing 0.05% Tween-20 (PBST) and blocked overnight at 4°C with 200 μL per well of 2% BSA in PBS. After washing 3 times with PBST, serial dilutions of humanized CD276 mAb (0–4 μg/mL for human antigen, 0–24 μg/mL for monkey and mouse antigens, and 0–60 μg/mL for rat antigen) were added and incubated for 1 hour at 37°C. Plates were then washed 5 times with PBST and incubated with horseradish peroxidase (HRP)–conjugated anti-human IgG secondary antibody (50 ng/mL; Cell Signaling Technology, cat. #32935) for 30 minutes at 37°C. Then, 100 μL of TMB substrate (cat. #34021, Thermo Fisher Scientific) was added to develop color for 10 minutes at 37°C. The reaction was terminated by adding 100 μL of 1 N H_2_SO_4_, and absorbance was measured at 450 nm using a SpectraMax iD3 plate reader (Molecular Devices).

### Generation of DualADCs and single-payload ADCs

Most linkers, free drugs, and linker–drug conjugates were dissolved in dimethyl sulfoxide at a final concentration of 10 mmol/L and stored at −20°C for long-term use. The cross-linker sulfosuccinimidyl 4-(N-maleimidomethyl) cyclohexane-1-carboxylate (sulfo-SMCC, Thermo Fisher Scientific, cat. #A39268) solution was prepared at a concentration of 22.9 mmol/L using deionized water prior to use. The reducing agent tris(2-carboxyethyl)phosphine (TCEP) hydrochloride was dissolved in deionized water at a final concentration of 5 mmol/L (pH 7.0) and was stable at room temperature for up to 2 weeks. The DualADC construction was performed at 1 to 20 mL scales with a final concentration of 2 or 5 mg/mL. PBS was used as the conjugation buffer for all ADCs in this study.

#### Conjugation of mAb-DM1/IMQ

The 10 mmol/L stock of mertansine (DM1, MedChemExpress, cat. #HY-19792) and mAb conjugation via lysine was performed by adding freshly prepared sulfo-SMCC (22.9 mmol/L) and DM1 to mAb (2 mg/mL) at a molar ratio of 18.2:14:1 (DM1/linker/mAb) and incubated at room temperature for 30 minutes. The mAb-DM1 ADC was purified using a protein A column, desalted via buffer exchange, and concentrated in PBS. Then, mAb-DM1 (5 mg/mL) was fully reduced using TCEP at a molar ratio of 44:1 (TCEP/mAb-DM1) by incubating at 37°C for 30 minutes and cooling at room temperature. The rebridging cleavable linker dibromomaleimide (DBM)-Val-Cit-PABC-PNP was synthesized with the TLR 7/8 dual agonist 1 imidazoquinoline (IMQ) to generate DBM-Val-Cit-PABC-PNP-IMQ using a custom design service (MedChemExpress). Then DBM-Val-Cit-PABC-PNP-IMQ was added to the fully reduced mAb-DM1 at a molar ratio of 20:1 (linker-drug/mAb), and the mixture was incubated at room temperature for 1 hour to completely rebridge mAb and synthesize mAb-DM1/IMQ. Finally, DualADC was purified using a protein A column, desalted, concentrated, and stored at 4°C or −20°C for *in vitro* and *in vivo* evaluations.

#### Conjugation of mAb-DXd/IMQ

First, the mAb-DXd was constructed by adding a 10 mmol/L stock solution of maleimidocaproyl (MC)-GGFG-DXd (MedChemExpress, cat. #HY-13631E) to the fully reduced mAb (5 mg/mL) prepared with TCEP as described above (TCEP/mAb molar ratio of 44:1, MC-GGFG-DXd/mAb molar ratio of 14:1), followed by incubation at room temperature for 1 hour and purification using a protein A column. Next, NHS-Phosphine (Thermo Fisher Scientific, cat. #88900) was added to the purified mAb-DXd at a molar ratio of 14:1 (NHS-Phosphine/mAb-DXd) in reaction A. In a separate reaction B, NHS-Azide (Thermo Fisher Scientific, cat. #88902) and IMQ were mixed at a molar ratio of 28:44.8 (NHS-Azide/IMQ). Both reactions were incubated at 37°C for 1 hour. Finally, mAb-DXd/IMQ was generated by adding the reaction B mixture to the reaction A mixture and incubating at 37°C for 2 to 4 hours to ensure complete conjugation. The DualADC mAb-DXd/IMQ was purified using a protein A column, desalted, concentrated, and stored at 4°C or −20°C for *in vitro* and *in vivo* evaluations.

#### Conjugation of mAb-MMAF/IMQ (control)

The mAb-MMAF/IMQ conjugate was prepared following our previously established protocol (21) with modifications. Briefly, mAb-MMAF (5 mg/mL) was conjugated at cysteine residues at a molar ratio of 7:1 (DBM-MMAF/TCEP reduced mAb) and purified using a protein A column. Next, NHS-Phosphine was added to mAb-MMAF at a molar ratio of 14:1 (NHS-Phosphine linker/mAb-MMAF) in reaction A, and NHS-Azide and IMQ (28:44.8) were mixed in reaction B with 1-hour incubation at 37°C. Finally, reaction B was added to reaction A with incubation at 37°C for 2 to 4 hours to achieve maximal reaction rates. The DualADC mAb-MMAF/IMQ was purified using a protein A column, desalted, concentrated, and stored at 4°C or −20°C for *in vitro* and *in vivo* evaluations.

#### Conjugation of single-payload ADCs (controls)

Five single-payload ADCs, including mAb-DM1 at lysine, mAb-DXd at cysteine, mAb-MMAF at cysteine, mAb-IMQ at lysine, and mAb-IMQ at cystine, were constructed following the procedures described above or our established protocols (9, 20, 21, 37–39).

### Purification of ADCs

In the developed DualADC conjugation procedures, linkers and drugs were used in excess, resulting in mAb conjugation rates of 91% to 100%, as determined by characterization using ultra-performance liquid chromatography–electrospray ionization-tandem mass spectrometry (UPLC-ESI-MS/MS) and high-performance liquid chromatography (HPLC). Single-step protein A purification was employed to purify DualADCs, followed by ultrafiltration with a 10 or 100 kDa PES MWCO membrane, as described before (21). This approach achieved >99% purity of the end product when the mAb conjugation rate exceeded 99%.

### ADC characterizations

#### HPLC

A Shimadzu HPLC system was used to evaluate the conjugation efficiency of ADCs (39). Mobile phase A consisted of 1.5 mol/L (NH_4_)_2_SO_4_ and 50 mmol/L mol/L Na_3_PO_4_ at pH 7.0. Mobile phase B consisted of 50 mmol/L mol/L Na_3_PO_4_ at pH 7.0. ADC samples, with mAb as control, were analyzed using an HIC-Butyl HPLC Column (Thermo Fisher Scientific, cat. #088558) at a mobile phase flow rate of 1 mL/minute and 25°C. The conjugation rate of ADCs was calculated using the following equation:

$$\frac{\text{ADC}}{\text{ADC}\;+\;\text{mAb}}\;\times \;100\%$$ (A)

#### UPLC-ESI-MS/MS

The DARs and DDRs of single-payload ADCs and DualADCs were determined from the MS data collected using UPLC-ESI-MS/MS at Creative Proteomics (Shirley). Analyses were conducted on a Waters Xevo G2 QTOF mass spectrometer coupled with a Waters Acquity UPLC system (Waters). Sample separation was achieved using a 2.1 × 50 mm diphenyl column with a 2.7-μm particle size (HALO Columns). Mobile phase A comprised 0.05% trifluoroacetic acid (TFA) in HPLC-grade water, and mobile phase B comprised 0.05% TFA in acetonitrile. A linear gradient elution was applied: 10% to 20% B from 0 to 1 minute, 20% to 50% B from 1 to 8 minutes, and 50% to 70% B from 8 to 10 minutes. MS was performed in positive ion mode, with data collected over a range of 500 to 6,000 m/z using MassLynx software (version 4.2). Spectra were deconvoluted using ProMass software for MassLynx version 3.0. The homogeneity of DualADCs and single-payload ADCs was evaluated using the DAR distribution profiles, as determined from UPLC-ESI-MS/MS data. Average DAR was calculated as follows:

$$\text{DA}R_{\text{ave}}\;=\;\frac{\sum \left({\text{inte}n\text{sity}}_{i}\;\times \;\text{payload}\;{\text{number}}_{i}\right)}{\sum {\text{intensity}}_{i}}$$ (B)

where intensity_*i*_ represents the peak intensity of ADC_i_ with DAR_i_. DDR was used to estimate the relative therapeutic function of chemotherapy and immunotherapy in the tumor microenvironment. DDR was calculated as follows:

$$\text{DDR}=\frac{{\text{DAR}}_{a}}{{\text{DAR}}_{b}}$$ (C)

where DAR_a_ is the potent payload-to-antibody ratio and DAR_b_ is the immune-boosting reagent-to-antibody ratio.

### Tumor targeting and drug delivery of DualADCs

#### Flow cytometry

Following the established protocol from our previous publications (9, 20, 21, 37, 38, 40), flow cytometry analysis was performed to evaluate the surface receptor binding of humanized anti-CD276 mAb-derived DualADCs. Briefly, 1 × 10^6^ TNBC cells were harvested and resuspended in 100 μL of flow cytometry buffer (1% FBS in PBS). Approximately 1 μg of DualADCs or mAbs labeled with Alexa Fluor 647 (AF647; Thermo Fisher Scientific, cat. #A88068) was added to the suspended cells. After incubation at 37°C for 30 minutes (human cell lines) or 1 hour (rat and mouse cell lines), the stained cells were washed 3 times with 1 mL of flow cytometry buffer and analyzed using a BD LSRFortessa flow cytometer (BD Biosciences) at the university core facility. Data were processed and analyzed using FlowJo software, with gating set to <0.5% fluorescent population in cells without mAb and ADC staining.

#### Live-cell confocal microscopy

Confocal microscopy imaging was performed to visualize the internalization of DualADCs in cancer cells according to our previously reported protocols (9, 20, 21, 37, 38, 40). A total of 1 × 10^6^ MDA-MB-468-FLuc-GFP cells were seeded in 35 mm glass-bottom dishes with 2 mL of medium and incubated overnight. Then, 20 μg of DualADCs labeled with Cy5.5 (Lumiprobe, cat. #7321) was added and incubated at 37°C for 20 hours to allow internalization. Confocal microscope images were acquired to validate DualADC internalization in TNBC cells.

#### *In vivo* imaging system

The live-animal *in vivo* imaging system (IVIS) was used to assess the *in vivo* biodistribution and tumor targeting of DualADC. Mice bearing 50 to 100 mm^3^ human TNBC xenografts were intravenously injected with 240 μg of Cy5.5-labeled DualADC via the tail vein. At 24 hours after injection, D-luciferin was intraperitoneally injected, and both bioluminescent and fluorescent images were acquired. Major organs (brain, heart, lung, spleen, kidneys) and tumors were harvested for *ex vivo* imaging to confirm DualADC distribution.

### *In vitro* cytotoxicity assay

The *in vitro* anticancer cytotoxicity of DualADCs was evaluated using three TNBC cell lines. Human TNBC MDA-MB-231 and MDA-MB-468 cells and mouse TNBC 4T1 cells were seeded in a 96-well plate at densities of 1,000, 10,000, and 500 cells/well in 200 μL of medium, respectively. Cells were treated with DualADCs, single-payload ADCs, or free drugs (controls) at concentrations of 0 to 200 nmol/L or 0 to 500 nmol/L for 5 days. Cell viability was measured using an MTT Cell Proliferation Assay kit according to the manufacturer’s instructions. Absorbance was recorded at 570 nm using a SpectraMax iD3 plate reader (Molecular Devices). Half-maximal inhibitory concentration (IC_50_) values were calculated using GraphPad Prism software (GraphPad).

### 4T1-FLuc xenografted syngeneic mouse models

To establish syngeneic models, 6-week-old female BALB/cJ mice (The Jackson Laboratory, cat. #000651, RRID: IMSR_JAX:000651) were subcutaneously injected with 2 × 10^6^ mouse TNBC 4T1-FLuc cells (*n* = 15/group). Mice with similar tumor volumes (20–50 mm^3^) and without ulceration were randomized into five groups (*n* = 6–8/group). Xenografted mice were treated with 16 mg/kg of mAb-DM1/IMQ, mAb-MMAF/IMQ, mAb-DXd/IMQ, mAb-DXd/IMQ in combination with anti-mouse PD-1 mAb-IMQ, or saline (control). ADCs were intravenously administered via the tail vein every 4 days for a 5-course schedule. Mice with tumor ulceration >2 mm were removed per early removal criteria. Tumor growth and body weight were measured twice a week. Tumor size was measured using digital calipers, and volume was calculated as (length × width^2^)/2. When tumor volume exceeded 1,000 mm^3^ in the control group, mice were euthanized for posttreatment analysis (*n* = 4/group). Tumors and major organs (heart, liver, spleen, lungs, kidneys) were harvested for posttreatment analysis, including histology analysis and bulk RNA sequencing (RNA-seq), as described below.

### MDA-MB-231-FLuc xenografted mouse models

Human TNBC xenografts were generated by subcutaneously implanting 5 × 10^6^ MDA-MB-231-FLuc cells into 6-week-old female Nude mice (J:NU HOM Homozygous for Foxn1<nu>, The Jackson Laboratory, cat. #007850, RRID: IMSR_JAX:007850). When tumor volumes reached 50 to 100 mm^3^, mice carrying similar tumor volumes were randomized into three groups (*n* = 6–8/group). Groups were treated with 16 mg/kg of mAb-DXd/IMQ, 16 mg/kg of mAb-DM1/IMQ, and saline, twice a week via tail vein injection. Tumor volume was measured by digital caliper and calculated as follows: (length × width^2^)/2. Mice were euthanized 30 days after treatment for posttreatment analysis (*n* = 4/group).

### IHC and hematoxylin and eosin staining

#### IHC staining

Normal organ tissue arrays from mouse (cat. #MO1601b), rat (cat. #RAT901b), and cynomolgus monkey (cat. #CyFDA1g) were purchased from TissueArray. Slides were baked at 60°C for 1 hour, deparaffinized in xylene, and rehydrated through graded ethanol. Blocking was performed with 3% hydrogen peroxide in Tris-buffered saline (TBS) for 10 minutes, followed by 5% normal goat serum (cat. #31872, Thermo Fisher Scientific) in TBS for 1 hour. Our humanized anti-CD276 mAb was diluted to 1 μg/mL in TBST containing 5% normal goat serum and applied to the array slides overnight at 4°C. After washing in TBST for 1 hour, goat anti-human IgG secondary antibody-HRP (cat. #31410, Thermo Fisher Scientific), diluted to 1 μg/mL in TBST with 5% normal goat serum, was added and incubated for 1 hour at room temperature. Following TBST washing, metal-enhanced DAB substrate (10×; cat. #34065, Thermo Fisher Scientific), diluted in stable peroxide buffer, was added for color development (10 minutes). Slides were counterstained with Mayer’s hematoxylin, dehydrated in ethanol and xylene, and mounted.

#### Hematoxylin and eosin staining

Hematoxylin and eosin (H&E) staining of paraffin-sectioned slides from harvested organs in animal studies was performed following previously described protocols (21, 37, 40–42).

### Bulk RNA-seq and data analysis

TNBC tumors were harvested from syngeneic models at the study endpoint. Total mRNA was isolated using the RNeasy Plus Mini Kit (QIAGEN). cDNA library construction and bulk RNA-seq were performed at Novogene America using Illumina HiSeq X Ten. Differentially expressed genes between the treatment and control groups were identified using the edgeR package (version 4.6.3) in R. Genes with a false discovery rate (FDR) <0.05 and absolute log_2_ fold change >0.58 (corresponding to a 1.5-fold change) were considered significantly differentially expressed. Multiple testing correction was performed using the Benjamini–Hochberg procedure to control the FDR.

### Statistical analysis

All quantitative data were presented as mean ± standard error of the mean, unless otherwise stated. Dose–response curves were fitted using nonlinear regression, and IC_50_ values were calculated with GraphPad Prism software (version 10.6.1, GraphPad Software) using the standard sigmoidal dose–response (variable slope) model. For comparisons between two independent groups, statistical significance was evaluated using an unpaired, two-tailed Student *t* test. When comparing multiple treatment groups with a single control group, a one-way analysis of variance was performed, followed by Dunnett’s *post hoc* test to identify significant differences from the control. A *P* value of < 0.05 was considered statistically significant.

## Results

### CD276 expression and mAb characterizations

**Figure 1.**
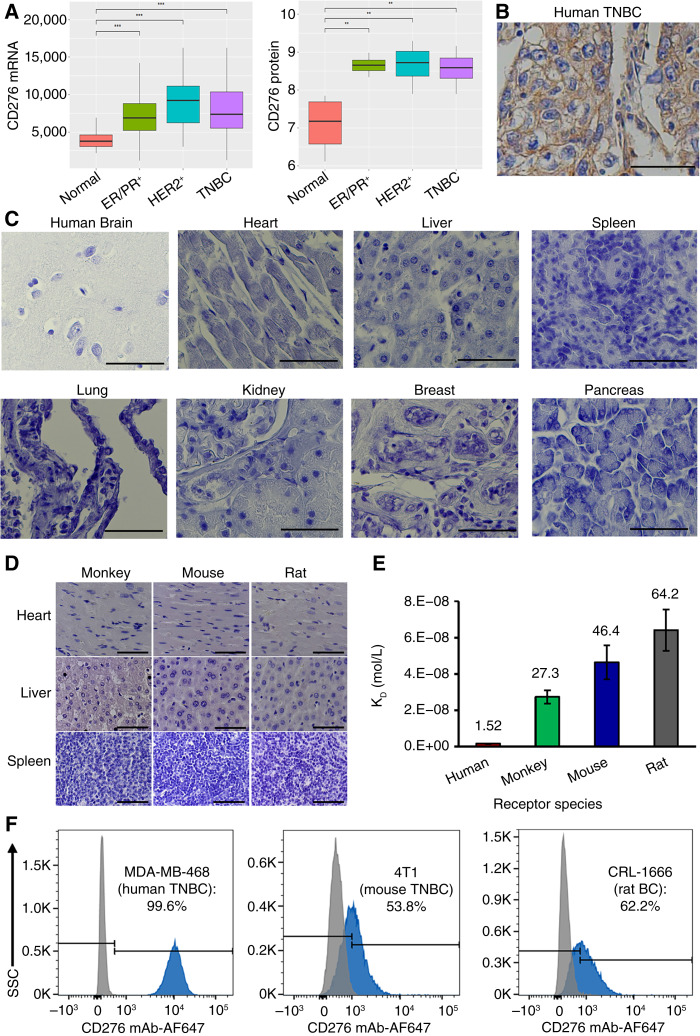
Characterization of CD276 expression and evaluation of humanized CD276 mAb binding and cross-species specificity. **A,** TCGA mRNA and protein analysis of CD276 expression in normal breast and breast cancers. **, *P* < 0.01; ***, *P* < 0.001. **B,** Representative image showing high CD276 expression in human TNBC tissue. Scale bar equals 70 μm. **C,** Representative IHC images of human normal organs stained with our humanized CD276 mAb. Scale bar equals 70 μm. **D,** Representative IHC images of monkey, mouse, and rat normal organs stained with humanized CD276 mAb. Scale bar equals 70 μm. **E,** Binding affinity (K_D_ values) of humanized CD276 mAb for CD276 receptors from human, monkey, mouse, and rat. **F,** Flow cytometry analysis of humanized CD276 mAb binding to the surface of human and mouse TNBC cells and rat breast cancer cells. SSC, side scatter.

Analysis of the TCGA-BRCA database revealed significantly higher transcript levels of CD276 in TNBC, ER^+^/PR^+^, and HER2^+^ breast cancers compared with normal breast tissue (Fig. 1A). Similarly, ProteomicsDB analysis indicated elevated CD276 protein expression in these breast cancer subtypes relative to normal breast tissue (Fig. 1A). IHC staining of TNBC tissues further confirmed strong membrane expression of CD276 (Fig. 1B). Accordingly, we developed and humanized an anti-CD276 mAb to target TNBC (21) and other cancers (20).

IHC staining of normal human tissues (brain, heart, liver, spleen, lung, kidney, breast, and pancreas) demonstrated no or minimal off-target binding by our humanized CD276 mAb (Fig. 1C). IHC staining of heart, liver, and spleen tissues from monkey, mouse, and rat using our humanized mAb also showed minimal binding (Fig. 1D). Importantly, affinity analysis yielded K_D_ values of 1.5 nmol/L for human CD276, 27.3 nmol/L for monkey, 46.4 nmol/L for mouse, and 64.2 nmol/L for rat (Fig. 1E). The single-digit KD value for the human receptor indicated that our humanized anti-CD276 mAb is well suited for delivering potent payloads to tumors with high CD276 expression. These results also demonstrated cross-species reactivity, supporting the use of these species for toxicology studies. The relatively lower affinity for mouse and rat CD276 was attributable to the human-derived immunogen used in antibody development. Flow cytometry analysis showed high surface binding rates for the anti-CD276 mAb in human TNBC MDA-MB-468 cells (99.6%), moderate binding in mouse TNBC 4T1 cells (53.8%), and moderate binding in rat breast cancer MAT-B-III cells (62.2%; Fig. 1F). Collectively, these findings indicate that the CD276 mAb has good affinity, high cancer binding rates, minimal off-target effects, and cross-species reactivity, making it a promising vehicle for the targeted delivery of cytotoxic agents.

### Construction and characterization of DualADCs

#### Humanized CD276 mAb

The volumetric productivity of the humanized anti-CD276 mAb reached 60 mg/L in a 2-L fed-batch bioreactor (Supplementary Fig. S1A). The mAb was purified through protein A affinity chromatography using a liquid chromatography system (Supplementary Fig. S1B).

#### New DualADCs conjugation

**Figure 2.**
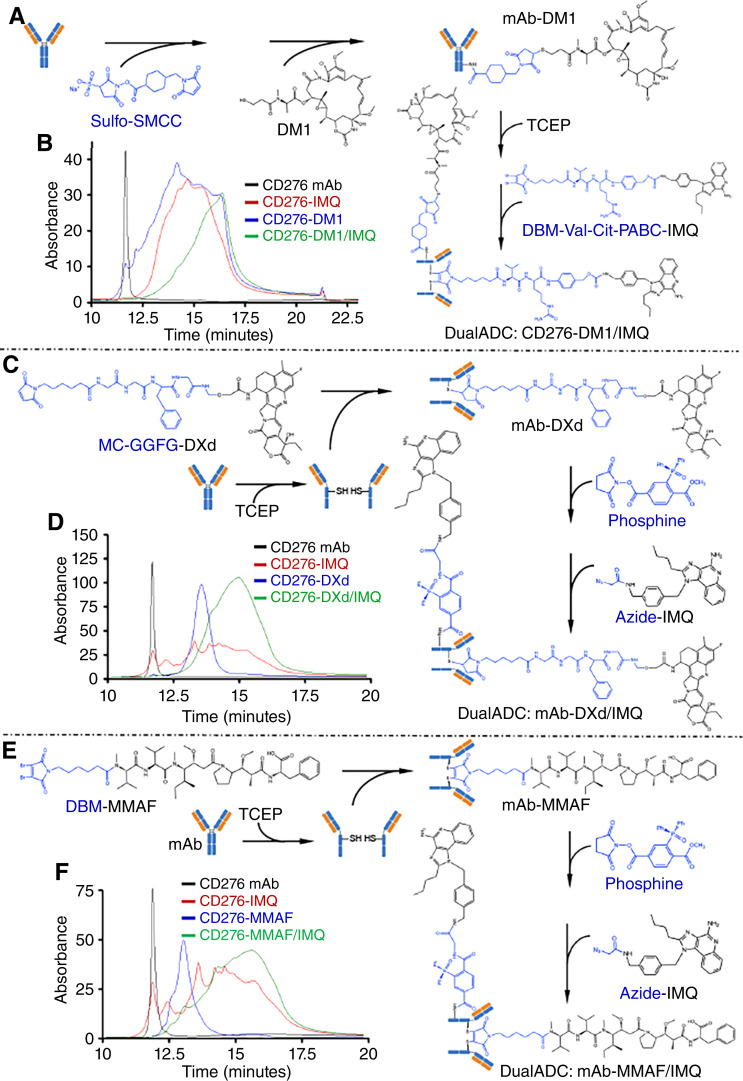
Schematic illustration of DualADC conjugation strategies and HPLC characterization. **A,** mAb-DM1/IMQ: conjugation of DM1 to the mAb via a lysine-based strategy, followed by conjugation of IMQ to the TCEP-reduced mAb-DM1 ADC via a cysteine-based strategy. **B,** HPLC characterization confirming the conjugation of mAb-DM1/IMQ. **C,** mAb-DXd/IMQ: conjugation of DXd to the TCEP-reduced mAb via a cysteine-based strategy, followed by conjugation of IMQ via a lysine-based strategy. **D,** HPLC characterization confirming the conjugation of mAb-DXd/IMQ. **E,** mAb-MMAF/IMQ: conjugation of MMAF to the TCEP-reduced mAb via a cysteine-based strategy, followed by conjugation of IMQ to the mAb-MMAF ADC via a lysine-based strategy. **F,** HPLC characterization confirming the conjugation of mAb-MMAF/IMQ.

**Figure 3.**
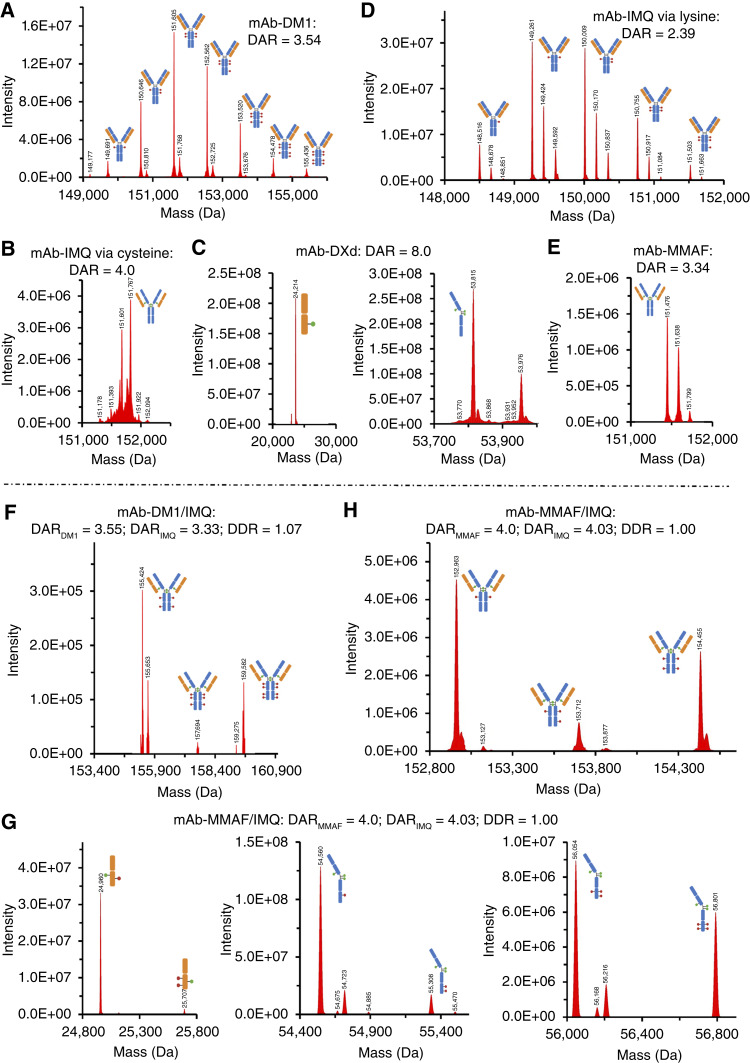
DAR determination and MS spectra of single ADCs and DualADCs using UPLC-MS. **A–E,** MS spectra of single ADCs: (**A**) mAb-DM1 ADC (DAR = 3.54). **B,** mAb-IMQ ADC via cysteine (DAR = 4). **C,** mAb-DXd ADC (DAR = 8). **D,** mAb-IMQ ADC via lysine (DAR = 2.26). **E,** mAb-MMAF ADC (DAR = 3.34). **F–H,** MS spectra of DualADCs: (**F**) mAb-DM1/IMQ with DAR_IMQ_ = 3.33, DAR_DM1_ = 3.55, and DDR = 1.07; (**G**) mAb-MMAF/IMQ with DAR_MMAF_ = 4.0, DAR_IMQ_ = 4.03 and DDR = 1.00. (**H**) mAb-MMAF/IMQ with DAR_MMAF_ = 4, DAR_IMQ_ = 2.36, and DDR = 1.63.

Diagrams of DualADC conjugation procedures and HPLC-HIC characterizations were presented in Fig. 2A–F. ESI-MS/MS validation and DAR analysis for cysteine- or lysine-based conjugations of single-payload ADCs are described in Fig. 3A–E. ESI-MS/MS validation, DARs, and DDR analysis for two-site (cysteine and lysine) conjugation of DualADCs are presented in Fig. 3F–H. Additional characterizations using HPLC-HIC (Supplementary Fig. S2A–S2J), flow cytometry (Supplementary Fig. S3), ESI-MS/MS (Supplementary Fig. S4A–S4C), and UPLC (Supplementary Fig. S5A–S5J) are summarized in supplemental figures.

#### mAb-DM1/IMQ

As illustrated in Fig. 2A, DM1 was conjugated via sulfo-SMCC at lysine residues of humanized CD276 mAb to generate mAb-DM1 (DM1/sulfo-SMCC:mAb ratio of 18.2:14:1) in step 1, followed by protein A purification. Subsequently, the mAb-DM1 was conjugated with our novel preconjugated DBM-Val-Cit-PABC-IMQ linker at cysteine residues (IMQ/mAb-DM1 ratio of 20:1) to generate DualADC-DM1/IMQ. The intermediate single-payload mAb-DM1 was validated by HPLC-HIC (Fig. 2A; Supplementary Fig. S2A and S2B), UPLC (Supplementary Fig. S5C), and ESI-MS/MS (Fig. 3A), with mAb as control (Supplementary Figs. S4A and S5A). mAb-DM1 conjugation at lysine exhibited greater heterogeneity, with a conjugate rate of 96.9%. As shown in Fig. 3A and Table 1, DAR_DM1_ ranged from 1 to 8, with an average DAR of 3.54, though major (74.80%) conjugates had DARs of 2 to 4. IMQ conjugation via cysteine residues was confirmed by HPLC-HIC (Fig. 2A; Supplementary Fig. S2C and S2D), UPLC (Supplementary Fig. S5D), and ESI-MS/MS (Fig. 3B). HPLC indicated the cysteine-based mAb-IMQ conjugation rate of 99.9% (Supplementary Fig. S2D), and ESI-MS/MS revealed DAR_IMQ_ of 4 with a homogeneous structure (Fig. 3B). DualADC mAb-DM1/IMQ was first characterized by HPLC with single-payload ADCs as controls (Fig. 2B) and HPLC (Supplementary Fig. S5H), showing a dual-payload conjugation rate of >99.6%. ESI-MS/MS validated two-site (cysteine and lysine) conjugation of dual-payload DM1/IMQ, with DAR_DM1_ of 3.55, DAR_IMQ_ of 3.33, and DDR_DM1/IMQ_ of 1.07 (Fig. 3F).

**Table 1.** DAR distribution, average DAR, and DDR of single-payload ADCs and DualADCs.

ADCs	DAR distribution	Average DAR	DDR
mAb-IMQ (via cysteine)	DAR 4: 100%	4	N/A
mAb-DXd (via cysteine)	Light chain: DAR 1: 100% Heavy chain: DAR 3: 100%	8	N/A
mAb-MMAF (via cysteine)	DAR 2: 1.49% DAR 3: 62.90% DAR 4: 35.61%	3.34	N/A
mAb-DM1 (via lysine)	DAR 0: 0.19% DAR 1: 2.79% DAR 2: 20.49% DAR 3: 30.72% DAR 4: 23.59% DAR 5: 13.26% DAR 6: 6.06% DAR 7: 2.01% DAR 8: 0.89%	3.54	N/A
mAb-IMQ (via lysine)	DAR 0: 0.48% DAR 1: 20.51% DAR 2: 35.99% DAR 3: 29.62% DAR 4: 10.67% DAR 5: 2.74%	2.39	N/A
mAb-DM1/IMQ	DAR 1 IMQ + 3 DM1: 15.37% DAR 2 IMQ + 2 DM1: 10.25% DAR 4 IMQ + 2 DM1: 29.85% DAR 4 IMQ + 3 DM1: 12.64% DAR 4 IMQ + 4 DM1: 4.16% DAR 4 IMQ + 5 DM1: 11.01% DAR 4 IMQ + 6 DM1: 0.17% DAR 4 IMQ + 7 DM1: 14.68% DAR 4 IMQ + 8 DM1: 1.87%	DAR_DM1_: 3.55 DAR_IMQ_: 3.33	1.07
mAb-DXd/IMQ	*Light chain (cleaved)*: DAR 1 DXd + 0 IMQ: 65.53% DAR 1 DXd + 1 IMQ: 31.19% DAR 1 DXd + 2 IMQ: 3.28% *Heavy Chain (cleaved)*: DAR 3 DXd + 0 IMQ: 32.68% DAR 3 DXd + 1 IMQ: 39.67% DAR 3 DXd + 2 IMQ: 19.52% DAR 3 DXd + 3 IMQ: 6.49% DAR 3 DXd + 4 IMQ: 1.50% DAR 3 DXd + 5 IMQ: 0.14%	DAR_DXd_: 8 DAR_IMQ_: 2.90	2.81
mAb-MMAF/IMQ	DAR 4 MMAF + 0 IMQ: 5.42% DAR 4 MMAF + 2 IMQ: 29.36% DAR 4 MMAF + 3 IMQ: 4.60% DAR 4 MMAF + 4 IMQ: 17.38% DAR 4 MMAF + 5 IMQ: 25.72% DAR 4 MMAF + 6 IMQ: 3.14% DAR 4 MMAF + 8 IMQ: 10.98%	DAR_MMAF_: 4 DAR_IMQ_: 4.03	1

Note: DDR is the molar ratio between highly potent payload and immune-boosting reagent.

#### mAb-DXd/IMQ

The TOP1 inhibitor DXd was preconjugated with a protease-cleavable GGFG motif and a MC linker to form the drug-linker MC-GGFG-DXd. This was conjugated to the reduced mAb (DXd/mAb ratio of 14:1) at 5 mg/mL in step 1 (Fig. 2C; Supplementary Figs. S2E and S5E) with mAb as control (Supplementary Figs. S4B, S4C, and S5B). Unlike DBM-based rebridging linkers, the MC maleimide group forms a single thioether bond per payload. ESI-MS/MS demonstrated a DAR_DXd_ of 8.0 (Fig. 3C), and HPLC-HIC confirmed a 99.9% conjugation rate for mAb-DXd (Fig. 2D; Supplementary Fig. S2F). Subsequently, IMQ was linked to mAb-DXd via a Phosphine-Azide linker at its lysine residues in step 2 (Fig. 2C; Supplementary Fig. S2G). ESI-MS/MS, HPLC, and UPLC confirmed the conjugation of IMQ via lysine (Fig. 3D; Supplementary Figs. S2H and S5F). HPLC confirmed DualADC formation with a >99.5% conjugation rate (Fig. 2D). ESI-MS/MS validated successful DXd/IMQ conjugation, with DAR_DXd_ of 8, DAR_IMQ_ of 2.90, and DDR_DXd/IMQ_ of 2.76 (Fig. 3G). HPLC showed heterogeneity in lysine conjugation of IMQ (Fig. 2D; Supplementary Fig. S2H), as confirmed in UPLC analysis (Supplementary Fig. S5I).

#### mAb-MMAF/IMQ

Following our previously developed platform (21) with optimizations, mAb-MMAF was generated via a DBM linker at cysteine (DBM-linker-MMAF/mAb ratio of 7:1) in step 1 and then conjugated with IMQ via a Phosphine-Azide linker at the lysine of mAb-MMAF in step 2 (Fig. 2E). HPLC-HIC showed 99.9% conjugation for mAb-MMAF and >95% for mAb-MMAF/IMQ (Fig. 2F; Supplementary Fig. S2I), consistent with UPLC characterization (Supplementary Figs. S5G and S5J). ESI-MS/MS indicated a homogeneous structure for mAb-MMAF (Fig. 3E; Supplementary Fig. S2J) due to stable thioether bonds. IMQ conjugation at lysine was confirmed by HPLC (Fig. 2F). ESI-MS/MS validated two-site dual-payload conjugation, with DAR_MMAF_ of 4, DAR_IMQ_ of 4.03, and DDR_MMAF/IMQ_ of 1 (Fig. 3H).

### DualADCs homogeneity

Antibody–drug conjugation typically produces a heterogeneous population of ADCs with varying numbers of conjugated payloads. The homogeneity of our constructed DualADCs was assessed by determining DARs and DDR values of the two payloads that were quantified on a Waters Xevo G2 QTOF MS coupled with Acquity UPLC. UPLC separation resolved these conjugated species with different DARs, allowing for the quantification of the relative abundance of each species. Subsequent MS/MS analysis confirmed the number and identity of payloads (i.e., cytotoxic payload or IMQ), allowing for the calculation of DARs.

#### Single-payload ADCs

As summarized in Table 1, cysteine-conjugated ADCs exhibited higher homogeneity with narrow DAR distributions. Specifically, mAb-IMQ (cysteine) had a DAR of 4. mAb-DXd (cysteine) showed a consistent DAR of 8 (100% DAR 1 in the light chain; 100% DAR 3 in the heavy chain) by ESI-MS/MS. mAb-MMAF (cysteine) had 62.90% DAR 3 and 35.61% DAR 4, yielding an average DAR_MMAF_ of 3.34. In contrast, mAb-DM1 (lysine) had DARs ranging from 1 to 7 (2.79%, 20.49%, 30.72%, 23.59%, 13.26%, 6.06%, 2.01%), with an average DAR_DM1_ of 3.54. mAb-IMQ (lysine) showed DARs ranging from 1 to 5 (20.51%, 35.99%, 29.62%, 10.67%, 2.74%), with an average DAR_IMQ_ of 2.39. The DBM rebridging linkers produced homogeneous ADCs.

#### DualADCs

Three DualADCs were constructed in this study. mAb-DM1/IMQ showed greater heterogeneity due to lysine-based DM1 conjugation. Although IMQ conjugation was homogeneous, the DAR of DM1 distributed as 2 (40.10%), 3 (28.01%), 4 (4.16%), 5 (11.01%), and 6 to 8 (16.72%), yielding an average DAR_DM1_ of 3.55, DAR_IMQ_ of 3.33, and DDR_DM1/IMQ_ of 1.07 (Table 1). Representative mass spectra showed DAR pairs like 4:4, 4:6, and 4:8 (IMQ/DM1) in Fig. 3F. For mAb-DXd/IMQ, the average DAR_DXd_ was 8 (1/light chain, 3/heavy chain), DAR_IMQ_ was 2.90, and DDR was 2.76. Representative spectra included the light chain with DXd/IMQ ratios of 1:0 (65.53%), 1:1 (31.19%), and 1:2 (3.28%) and cleaved chain DXd/IMQ ratios of 3:0 (32.68%), 3:1 (39.67%), 3:2 (19.52%), 3:3 (6.49%), and 3:4 (1.50%; Table 1). mAb-MMAF/IMQ had homogeneous MMAF conjugation (DAR_MMAF_ of 4) and relatively heterogeneous IMQ (DAR_IMQ_ of 4.03), with MMAF/IMQ ratios of 4:0 (5.42%), 4:2 (29.24%), 4:3 (4.60%), 4:4 (17.38%), 4:5 (25.72%), 4:6 (3.14%), and 4:8 (10.98%), and an average DDR_MMAF/IMQ_ of 1. Representative spectra showed peaks for 4:2, 4:3, and 4:4 (MMAF/IMQ) in Fig. 3H. UPLC-ESI-MS/MS used in this study had higher resolution to calculate accurate DAR and DDR than previously established HPLC-HIC, MALDI-MS, and UV spectrometry (21). Additionally, the DAR for payloads conjugated at lysine residues exhibited a “low–high–low–high” distribution pattern in the DualADC, in contrast to the Gaussian distribution observed in single-payload ADCs (Table 1), which needs further investigation in the future.

### Cancer specificity and drug delivery of three DualADCs

#### Cancer surface binding

**Figure 4.**
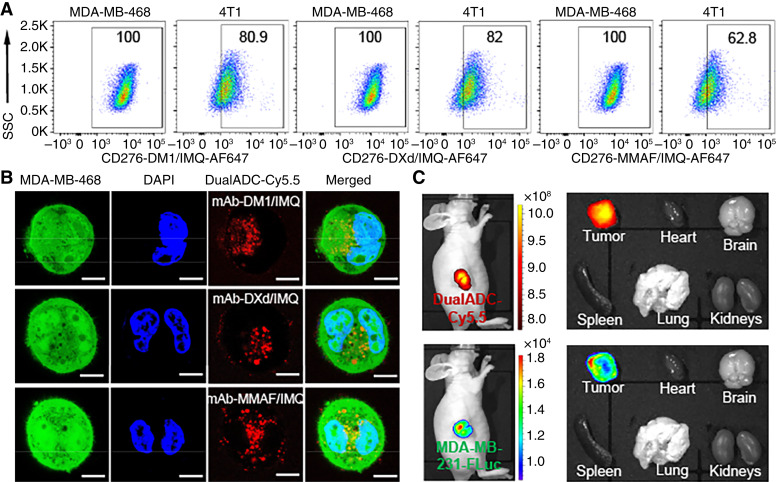
Evaluation of TNBC surface binding, internalization, and *in vivo* targeting of DualADCs. **A,** Flow cytometry analysis of DualADCs binding to the surface of human and mouse TNBC cells. **B,** Confocal microscopy images showing internalization of DualADCs-Cy5.5 in MDA-MB-468 cells. Scale bar, 10 μm. **C,** IVIS imaging of NSG mice xenografted with MDA-MB-231 tumors and harvested major organs following administration of DualADC (mAb-DXd-IMQ)-Cy5.5. SSC, saline sodium citrate.

Flow cytometry showed strong surface binding of all three humanized anti-CD276 mAb-derived DualADCs to human and mouse TNBC cell lines. mAb-DM1/IMQ, mAb-DXd/IMQ, and mAb-MMAF/IMQ exhibited binding rates of 99.9%, 100%, and 100% in human MDA-MB-231 cells, comparable with ∼100% for unconjugated mAb (Supplementary Fig. S3). All three DualADCs achieved 100% binding in human MDA-MB-468 cells versus 99.6% for mAb (Fig. 4A). In mouse 4T1 cells, binding rates were 80.9%, 82%, and 62.8%, respectively. Similar to mAb, the binding affinity to mouse TNBC was lower than the affinity to human TNBC but was still significant. These data indicate that dual-payload conjugation preserved cancer cell binding efficiency, suggesting that the conjugation reactions do not disrupt the structural integrity of DualADCs. Consistent with cross-species affinity data (Fig. 1E), binding to mouse TNBC was lower but substantial.

#### Internalization and drug delivery

The live-cell confocal microscopy revealed effective internalization of Cy5.5-labeled DualADCs (mAb-DM1/IMQ, mAb-DXd/IMQ, mAb-MMAF/IMQ) into TNBC MDA-MB-468-FLuc-GFP cells (Fig. 4B). DualADC fluorescence (red) overlapped with cancer cell cytoplasm (green) at 20 hours after incubation, confirming intracellular payload delivery.

#### *In vivo* tumor targeting

Live-animal IVIS imaging showed DualADC-Cy5.5 (red fluorescence) accumulation in TNBC tumor xenografts expressing FLuc (blue bioluminescence) at 24 hours after tail vein injection (Fig. 4C). *Ex vivo* imaging of harvested organs revealed minimal off-target accumulation in the heart, brain, spleen, lungs, and kidneys, with strong tumor accumulation. Overall, DualADCs demonstrated high surface binding, effective intracellular delivery, and *in vivo* tumor specificity, highlighting their potential for targeted payload delivery.

### Cytotoxicity of DualADCs

**Figure 5.**
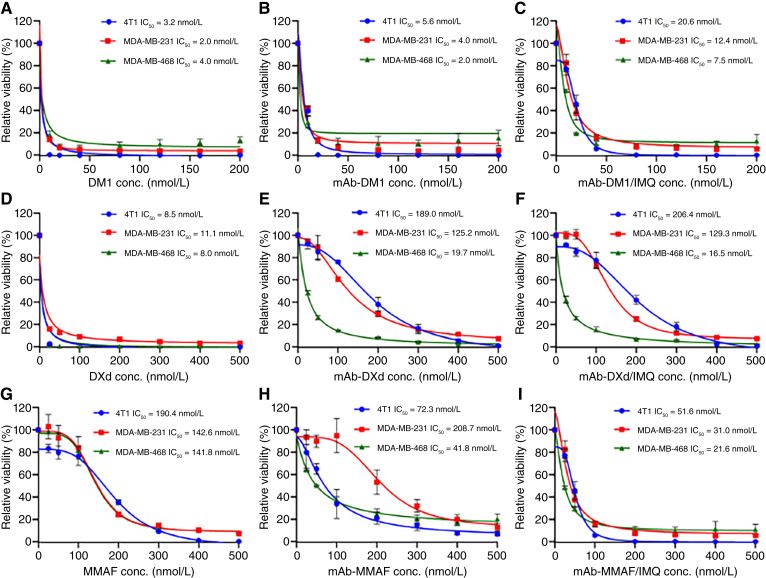
*In vitro* cytotoxicity evaluation of DualADCs. **A–C,** Cytotoxicity of free DM1, mAb-DM1 ADC, and mAb-DM1/IMQ in TNBC cells (*n* = 3). **D–F,** Cytotoxicity of free DXd, mAb-DXd ADC, and mAb-DXd/IMQ in TNBC cells (*n* = 3). **G–I,** Cytotoxicity of free MMAF, mAb-MMAF ADC, and mAb-MMAF/IMQ in mouse TNBC 4T1 cells and human TNBC MDA-MB-231 and MDA-MB-468 cells (*n* = 3).

Anticancer cytotoxicity of mAb-DM1/IMQ, mAb-DXd/IMQ, and mAb-MMAF/IMQ was assessed in human TNBC MDA-MB-231 and MDA-MB-468 cells, and mouse TNBC 4T1 cells, using free drugs and single-payload ADCs as controls. MTT assay results at drug concentrations of 0 to 200 nmol/L or 0 to 500 nmol/L are summarized in Fig. 5. The cytotoxicity assay results at low drug concentrations of 0 to 40 nmol/L were also performed, but only nine assays showed significant cell death, which is presented in Supplementary Fig. S6. mAb-DM1/IMQ had IC_50_ values of 6.4 to 12.4 nmol/L (MDA-MB-231), 7.5 to 9.2 nmol/L (MDA-MB-468), and 20.6 to 22.3 nmol/L (4T1), similar to or slightly higher than free DM1 (2–19.5, 4–10.9, 3.2–9.9 nmol/L) and mAb-DM1 (2.3–4, 2–6.5, 5.6–6.6 nmol/L; Fig. 5A–C; Supplementary Fig. S6A–S6C). mAb-DXd/IMQ showed IC_50_ values of 129.3 nmol/L (MDA-MB-231), 16.5 nmol/L (MDA-MB-468), and 206.4 nmol/L (4T1), similar to mAb-DXd (125.2, 19.7, 189 nmol/L) due to equivalent DAR_DXd_. Both were less potent than free DXd (11.1–11.6, 8–19.5, 7.5–8.5 nmol/L; Fig. 5D–F; Supplementary Fig. S6D), warranting further evaluation. mAb-MMAF/IMQ exhibited IC_50_ values of 31 nmol/L (MDA-MB-231), 21.6 nmol/L (MDA-MB-468), and 51.6 nmol/L (4T1), lower than mAb-MMAF (208.7, 41.8, 72.3 nmol/L) and free MMAF (142.6, 141.8, 190.4 nmol/L; Fig. 5G–I).

### Anticancer efficacy of DualADCs in syngeneic models

**Figure 6.**
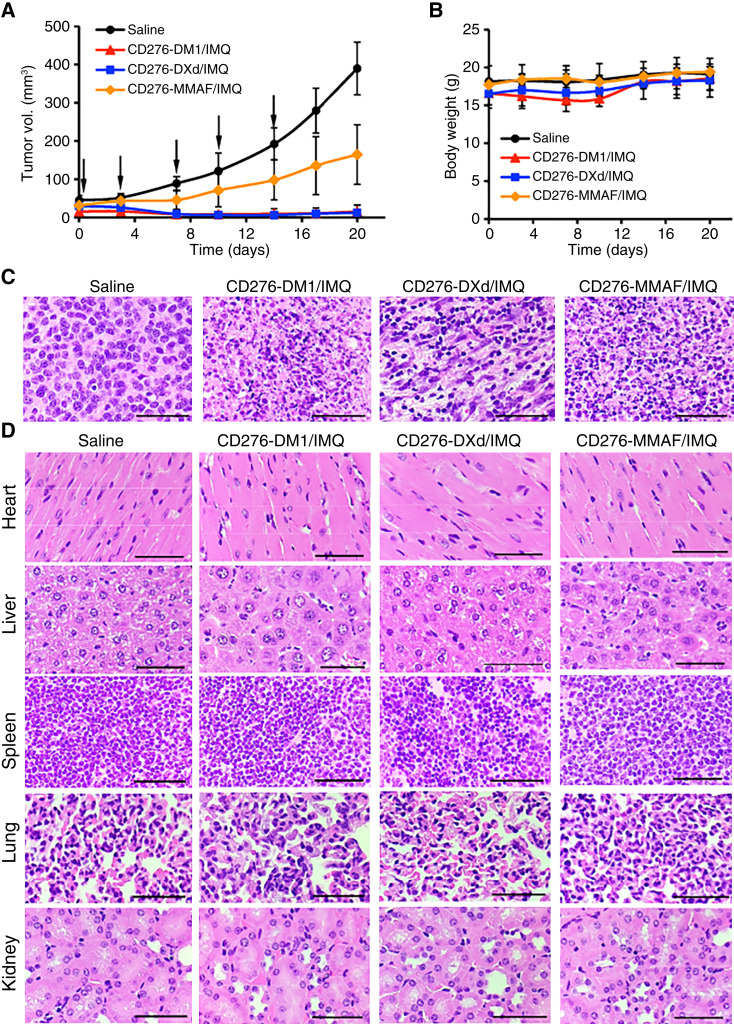
*In vivo* anti-TNBC efficacy of DualADCs in 4T1-Fluc xenograft immunocompetent mouse models. ADCs or saline (16 mg/kg) were i.v. injected via the tail vein. **A,** Tumor volume measurement with black arrows indicating treatments or saline (*n* = 4). **B,** Body weight. **C,** H&E staining of tumor tissues. Scale bar equals 70 μm. **D,** H&E staining of major organs (heart, liver, spleen, lung, and kidney) collected at the endpoint of the *in vivo* study. Scale bar equals 70 μm.

Therapeutic efficacy of the three DualADCs was evaluated in 4T1-FLuc xenografted BALB/cJ mice at 16 mg/kg (every 4 days for a 5-course schedule). All treatments significantly inhibited tumor growth versus saline, with mAb-DM1/IMQ and mAb-DXd/IMQ completely blocking tumor growth (Fig. 6A). At endpoint (ulceration >2 mm early removal criteria), the average tumor volumes were 12.66 mm^3^ (mAb-DXd/IMQ) and 15.58 mm^3^ (mAb-DM1/IMQ) versus 389.79 mm^3^ (saline) and 164.70 mm^3^ (mAb-MMAF/IMQ; Fig. 6A). No significant body weight differences were observed across groups (Fig. 6B). H&E staining confirmed extensive TNBC cell death in DualADC groups, with viable cells persisting in the saline control (Fig. 6C). H&E staining of major organs (brain, heart, liver, spleen, lungs, kidneys) showed no inflammation, necrosis, apoptosis, or damage in treatment groups (Fig. 6D), indicating manageable toxicity at the DualADC dose of 16 mg/kg following an injection schedule of every 4 days for 5 courses. Therefore, mAb-DM1/IMQ and mAb-DXd/IMQ were selected as promising candidates for further toxicity evaluation in human TNBC xenograft models.

The TNBC tumors that were treated with mAb-DXd/IMQ and saline (control) were harvested to perform bulk RNA-seq analysis (Supplementary Fig. S7). Multiple immune response pathways were upregulated by DualADC, including T-cell proliferation, selection, and activation; CD4^+^ T-cell proliferation; B-cell activation and proliferation; immunoglobulin production; leukocyte activation and mediated immunity; and lymphocyte proliferation and activation. This result confirmed the upregulation of tumoral immunity by DualADC.

### Anticancer efficacy of DualADCs in human TNBC xenograft models

**Figure 7.**
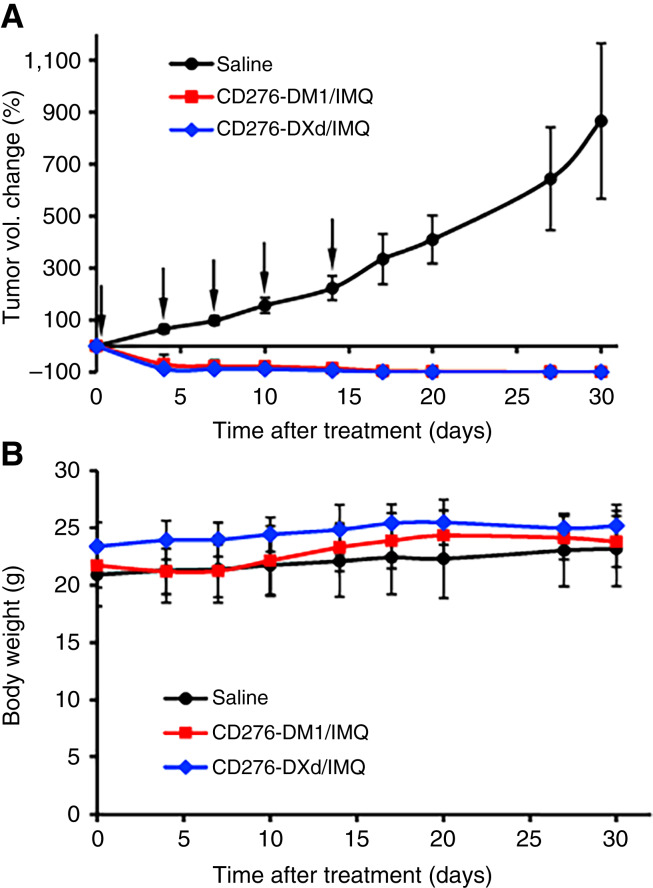
*In vivo* anti-TNBC efficacy of DualADCs in MDA-MB-231-FLuc xenograft immunocompromised mouse models. ADCs (16 mg/kg) were i.v. injected via the tail vein. **A,** Tumor volume measurement with black arrows indicating treatments or saline (*n* = 4). **B,** Body weight.

Antitumor efficacy of DualADCs was further validated in human TNBC xenografted mouse models. Female nude mice bearing MDA-MB-231-Fluc tumors were treated with mAb-DXd/IMQ or mAb-DM1/IMQ at a dose of 16 mg/kg, with saline as control. DualADCs were injected through the tail vein twice a week. Both DualADCs completely regressed human TNBC xenograft tumors without regrowth for 16 days after stopping treatment (Fig. 7A). Body weights had no drop throughout the treatment period (Fig. 7B), indicating tolerable toxicity. There were no tumor samples available in the treatment groups at the end for histology and other posttreatment analyses. These results demonstrate that our newly developed DualADCs in this study are highly effective against aggressive TNBC cancers.

## Discussion

In this study, we developed and characterized two novel DualADCs using a humanized anti-CD276 mAb for the treatment of TNBC. Unlike conventional single-payload ADCs, our DualADCs combine cytotoxic chemotherapy, such as well-established agents DM1, DXd, and MMAF in this study (and SN-38, MMAE, and PBD dimer in ongoing work), with an immune-boosting reagent, such as the TLR 7/8 agonist IMQ. This design for targeted chemoimmunotherapy enhances ADC efficacy against aggressive, heterogeneous, and/or drug-resistant cancers by integrating synergistic mechanisms: direct killing of cancer cells and tumoral immune activation within a single molecule. The targeted delivery of dual payloads via our humanized and engineered mAb, which exhibits excellent drug delivery properties, addresses key challenges of standard chemotherapy, including severe adverse effects, limited specificity, narrow therapeutic windows, and the development of drug resistance.

Besides our previous DualADC published in 2024 (21), several different DualADCs have been developed as advancements over conventional ADCs. For example, Yamazaki and colleagues (22) engineered an anti-HER2 mAb-derived ADC in 2021 by linking two similar cytotoxic payloads, namely, MMAF and MMAE. This construction relies on single specific-site conjugation and incorporates two potent drugs with a ratio of 1:1. Recently, McKertish and Kayser (23) conjugated two microtubule inhibitors (DM1 and MMAE) to an anti-HER2 mAb using cysteine and lysine residues in 2023, whereas Wilski and colleagues (43) linked MMAF and the TOPI inhibitor SN-38 with one mAb in 2025. However, neither of these two recent studies provides full characterization, validation, and evaluation.

A key innovation of our DualADC platforms, compared with these reported DualADCs, lies in the two-site (cysteine and lysine residues) conjugation, achieved through optimized procedures and novel linker designs, enabling control of DARs and DDR. Mass spectrometry confirmed efficient conjugation, with average DAR values of 2 to 4 for IMQ and 3 to 8 for cytotoxic payloads. Our conjugation technologies allow rational selection of potent payloads and immune regulators to achieve synergistic effects, with the capability of optimizing DARs and DDRs to maximize anticancer efficacy. Cysteine conjugation via the DBM linker yielded homogeneous ADCs. Our custom linkers (Phosphine-Azide and cleavable DBM-Val-Cit-PABC) for TLR agonist conjugation provide flexibility for dual-payload designs. Another advantage is that our all-in-one chemo-immunotherapeutic DualADCs incorporate a humanized anti-CD276 mAb that blocks immune checkpoints, a TLR agonist that engages innate and adaptive immunity, and cytotoxic payloads with diverse anticancer mechanisms. Unlike those studies, we conducted comprehensive characterizations using multiple analytic methods to validate dual-payload conjugation, accurately determining DARs and DDRs for all DualADCs and single-payload ADCs, which enable the tuning of DDR between the two drugs. Our optimized protocols achieved a high mAb conjugation efficiency of 91 to 99+%.

The favorable pharmacologic performance of our three DualADCs was validated both *in vitro* and *in vivo*. These DualADCs exhibited high cancer specificity, appropriate affinity, targeted payload delivery, potent anticancer cytotoxicity, and high therapeutic efficacy in mouse models. For instance, all three DualADCs demonstrated nanomolar IC_50_ values against human and murine TNBC cell lines. In contrast to our prior single-payload ADCs, these DualADCs significantly suppressed or eliminated tumor growth in both immunocompetent and immunocompromised models. Toxicity appeared minimal, as evidenced by the absence of systemic effects, body weight loss, or organ damage. Codelivery of cytotoxic and immune-boosting payloads was confirmed by bulk RNA-seq of tumor samples from animal studies, which revealed upregulation of multiple immune response pathways in the tumor microenvironment.

Despite these promising results, several challenges remain. First, this study evaluated DualADC efficacy using tumor xenografts. Our DualADCs combine the cancer-targeting capability of the mAb, direct cytotoxicity from DXd, DM1, or MMAF, and tumoral immunity upregulation by the TLR agonist, which could address advanced cancer proliferation and resistance. Thus, we plan to develop distant and spontaneous metastatic models in humanized mice for further assessment in the future. Second, only one therapeutic dose (16 mg/kg) was tested here, as our prior pharmacokinetics and tolerability studies on the control DualADC conjugated with MMAF and IMQ demonstrated high circulatory stability (t_1/2_ = 1.21–2.99 days, C_max_ = 21.39–93.37 μg/mL, D = 6.48–16.66 mg/kg, and τ = 5.13–7.10 days) and a safety margin of up to 32 mg/kg in mouse models (21). Future studies will examine efficacy at lower doses (4–16 mg/kg) and toxicity at higher doses (16–40 mg/kg) to define a safe and effective therapeutic window. We can leverage our mAb’s cross-species reactivity (human, mouse, rat, monkey) by using rat syngeneic models with rat breast cancer xenografts for toxicology, pharmacokinetics, and pharmacodynamics studies, alongside commercial human organoids for toxicity testing. Third, linker and drug circulation stability will be assessed in mouse models and human serum, with an advanced pharmacokinetics model developed to analyze data and predict optimal dosing and scheduling. Fourth, cancer-targeted delivery and potential bystander effects in the tumor microenvironment for both payloads will be investigated using advanced analytics (e.g., UPLC-ESI-MS/MS) in the future. Fifth, we will explore limiting steps and key parameters for large-scale DualADC conjugation in stirred-tank bioreactors. Kinetics models will simulate dual-payload conjugation, identify scale-up factors, and examine interactions between two-site conjugations, aiming for high rates and yields, minimal precipitation, greater homogeneity, and balanced cost–quality. Large-scale conjugations will validate these models. Finally, we plan to collaborate with machine learning experts to develop an AI model for accurate DualADC structure analysis and conjugation heterogeneity prediction.

In summary, we present a novel class of CD276-targeted DualADCs that integrate chemotherapy and immunotherapy into a single molecule. These DualADCs demonstrate potent antitumor activity, selective tumor targeting, and minimal off-target toxicity in preclinical TNBC models. The combination of two payloads could improve the treatment benefits of heterogeneous cancers and potentially increase patient response rates. The robust and scalable DualADC conjugation procedures can be easily scaled up to pilot and large-scale production. Our three platforms can also be readily adapted to other mAbs, peptides, and drugs, representing promising next-generation ADCs for targeted anticancer chemo-immunotherapy.

## Supplementary Material

Figure S1 Figure S1 shows production and purification of humanized CD276 mAb.

Figure S2 Figure S2 shows single ADCs structure and HPLC characterization.

Figure S3 Figure S3 shows evaluation of cancer surface binding by DualADCs.

Figure S4 Figure S4 shows MS spectra of intact mAb and fully reduced mAb.

Figure S5 Figure S5 shows Ultra Performance Liquid Chromatography (UPLC) chromatogram used to identify peaks prior to subsequent MS analysis.

Figure S6 Figure S6 shows in vitro cytotoxicity evaluation at low drug concentrations.

Figure S7 Figure S7 shows bulk RNA-Seq analysis of immune functions in TME.

## Data Availability

The data generated in this study are available from the corresponding author upon request. The bulk RNA-seq data generated in this study are publicly available in the Gene Expression Omnibus with accession number GSE312957 at https://www.ncbi.nlm.nih.gov/geo/query/acc.cgi?acc=GSE312957. The transcript data of CD276 in TNBC in this study were obtained from the TCGA-BRCA dataset, and CD276 protein expression data were obtained from ProteomicsDB. The bulk RNA-seq reproductive code was deposited to Code Ocean at https://codeocean.com/capsule/6252809/tree/v1. All materials used in the study are commercially available, except for the anti-CD276 mAb and single-payload ADCs and DualADCs. The Nude (J:NU HOM Homozygous for Foxn1<nu>) and BALB/cJ female mice were purchased from The Jackson Laboratory.
