# Supplementary material for: Advanced CD276-Targeting Dual-Payload Antibody–Drug Conjugates for Cancer Therapy
Source: Cancer Res Commun. 2026 Apr 21;6(4):898–912. doi: 10.1158/2767-9764.CRC-26-0059 (PMC13099120; doi:10.1158/2767-9764.CRC-26-0059)
Supplement: Figure S1 — shows production and purification of humanized CD276 mAb. [file crc-26-0059_figure_s1_suppsf1.docx]

**
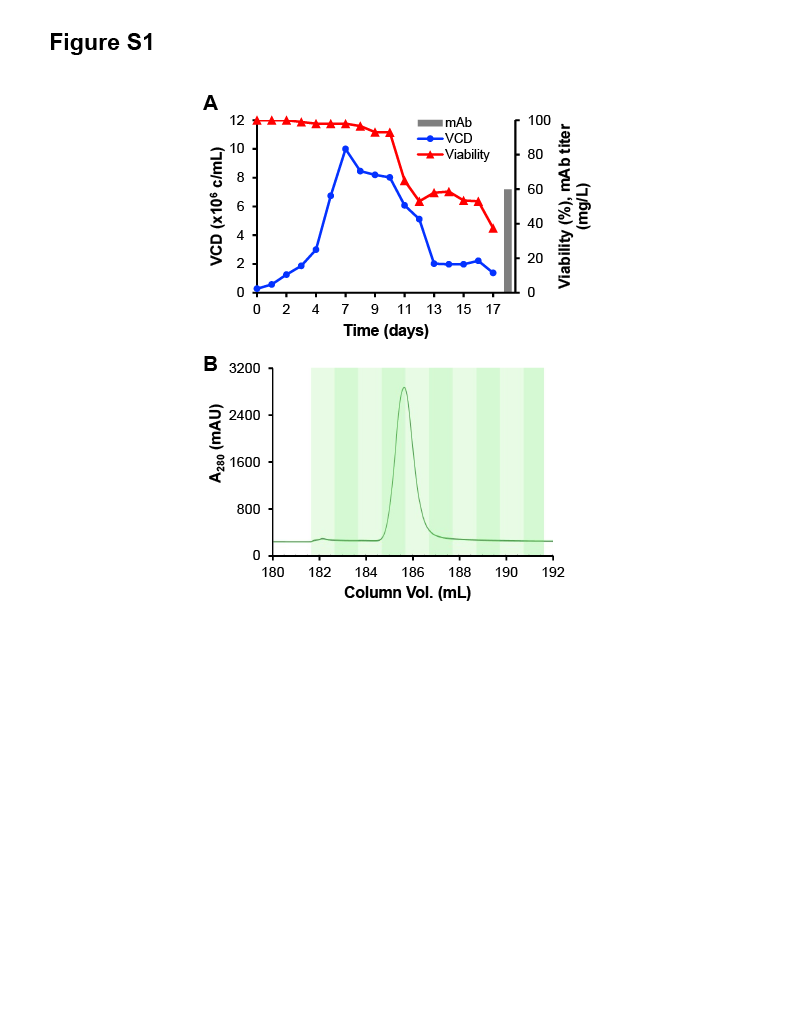
**

**Figure S1. Production and purification of humanized CD276 mAb.** (**A**) CD276 mAb production in CHO cells cultured in a 2-L bioreactor under controlled conditions (37 °C, 140 rpm, 40% DO, pH 7.2). (**B**) Purification of CD276 mAb using liquid chromatography equipped with protein A column.
