# Supplementary material for: Advanced CD276-Targeting Dual-Payload Antibody–Drug Conjugates for Cancer Therapy
Source: Cancer Res Commun. 2026 Apr 21;6(4):898–912. doi: 10.1158/2767-9764.CRC-26-0059 (PMC13099120; doi:10.1158/2767-9764.CRC-26-0059)
Supplement: Figure S2 — shows single ADCs structure and HPLC characterization. [file crc-26-0059_figure_s2_suppsf2.docx]

**
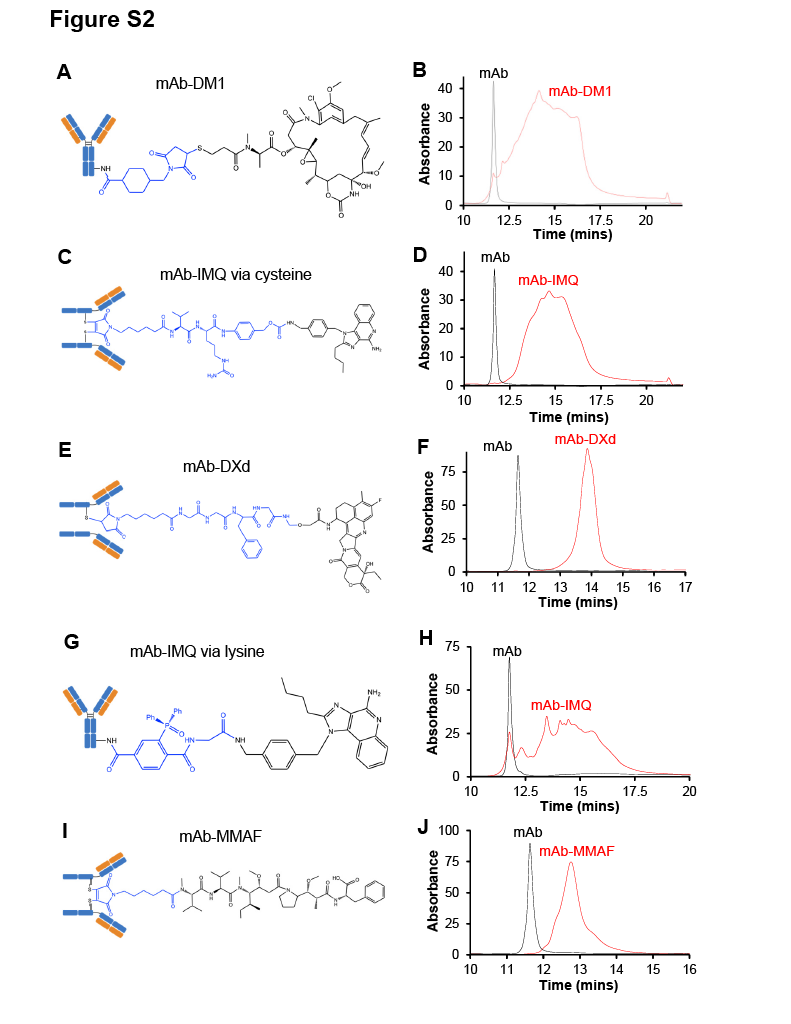
**

**Figure S2. Single ADCs structure and HPLC characterization.** (**A**) mAb-DM1 structure. (**B**) HPLC confirmation of mAb-DM1 conjugation. (**C**) mAb-IMQ ADC via cysteine structure. (**D**) HPLC confirmation of mAb-IMQ ADC via cysteine conjugation. (**E**) mAb-DXd ADC structure. (**F**) HPLC confirmation of mAb-DXd ADC conjugation. (**G**) mAb-IMQ ADC via lysine structure. (**H**) HPLC confirmation of mAb-IMQ ADC via lysine conjugation. (**I**) mAb-MMAF ADC structure. (**J**) HPLC confirmation of mAb-MMAF conjugation.
