# Supplementary material for: Advanced CD276-Targeting Dual-Payload Antibody–Drug Conjugates for Cancer Therapy
Source: Cancer Res Commun. 2026 Apr 21;6(4):898–912. doi: 10.1158/2767-9764.CRC-26-0059 (PMC13099120; doi:10.1158/2767-9764.CRC-26-0059)
Supplement: Figure S3 — shows evaluation of cancer surface binding by DualADCs. [file crc-26-0059_figure_s3_suppsf3.docx]

**
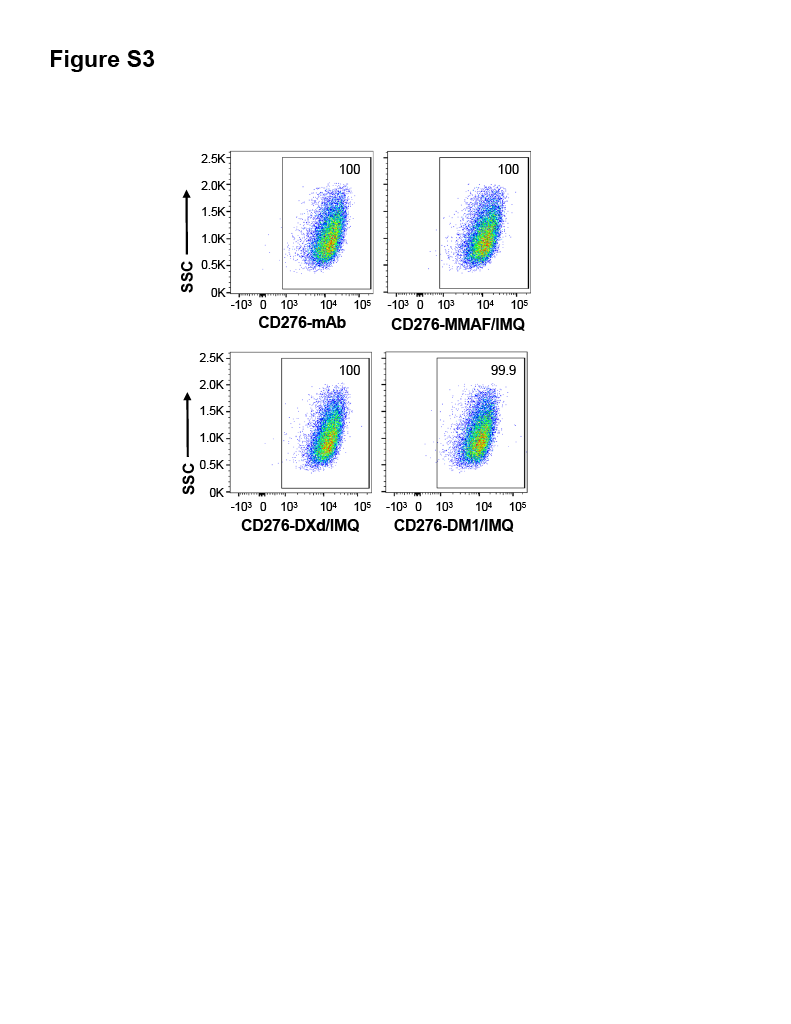
**

**Figure S3. DualADCs binding evaluation.** Flow cytometry analysis of mAb and DualADCs binding to the surface of human TNBC MDA-MB-231 cells.
