# Supplementary material for: Advanced CD276-Targeting Dual-Payload Antibody–Drug Conjugates for Cancer Therapy
Source: Cancer Res Commun. 2026 Apr 21;6(4):898–912. doi: 10.1158/2767-9764.CRC-26-0059 (PMC13099120; doi:10.1158/2767-9764.CRC-26-0059)
Supplement: Figure S4 — shows MS spectra of intact mAb and fully reduced mAb. [file crc-26-0059_figure_s4_suppsf4.docx]

**
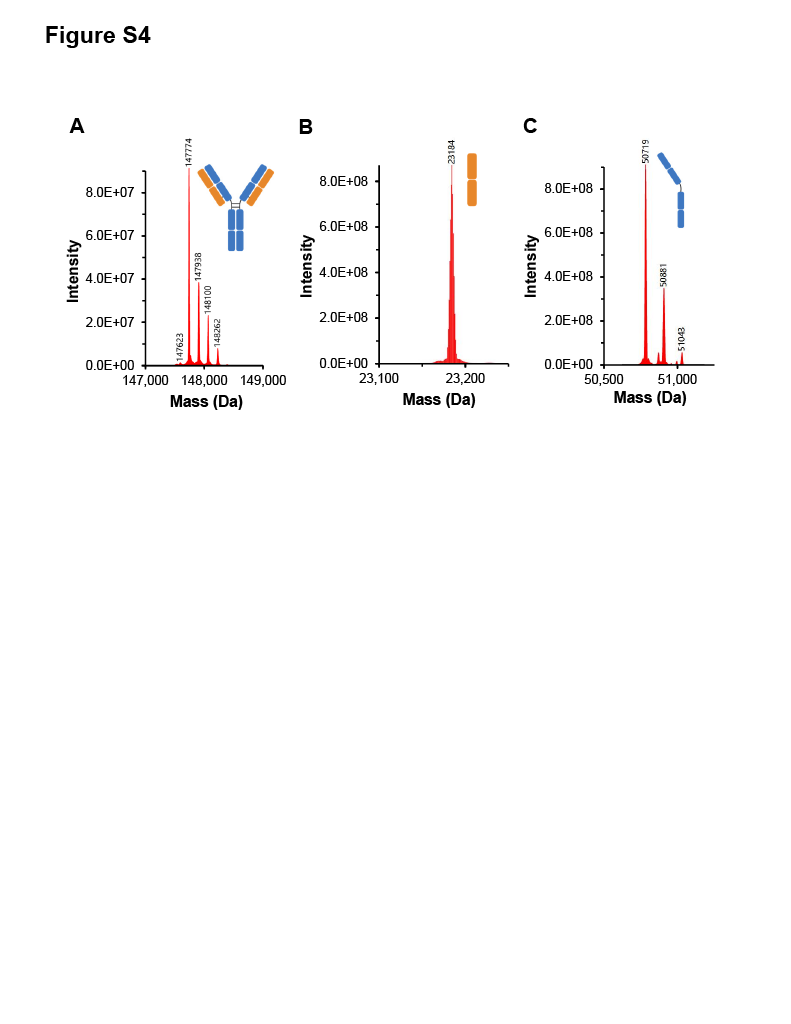
**

**Figure S4. MS spectra of intact mAb and fully reduced mAb.** (**A**) MS spectra of intact mAb. (**B**) MS spectra of reduced mAb light chain. (**C**) MS spectra of reduced mAb heavy chain.
