# Supplementary material for: Advanced CD276-Targeting Dual-Payload Antibody–Drug Conjugates for Cancer Therapy
Source: Cancer Res Commun. 2026 Apr 21;6(4):898–912. doi: 10.1158/2767-9764.CRC-26-0059 (PMC13099120; doi:10.1158/2767-9764.CRC-26-0059)
Supplement: Figure S5 — shows Ultra Performance Liquid Chromatography (UPLC) chromatogram used to identify peaks prior to subsequent MS analysis. [file crc-26-0059_figure_s5_suppsf5.docx]

**
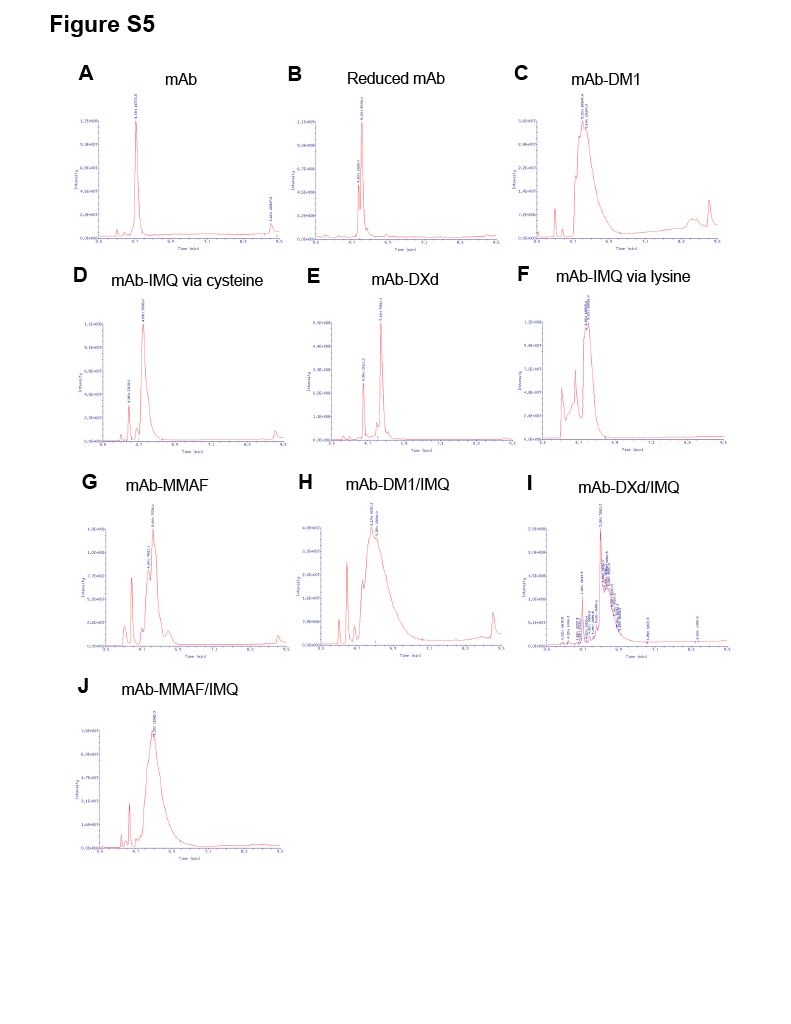
**

**Figure S5. Ultra performance liquid chromatography chromatogram used to identify peaks prior to subsequent MS analysis.** (**A**) Intact mAb. (**B**) Reduced mAb. (**C**) mAb-DM1 ADC. (**D**) mAb-IMQ ADC via cysteine ADC. (**E**) mAb-DXd ADC. (**F**) mAb-IMQ via lysine ADC. (**G**) mAb-MMAF ADC. (**H**) mAb-DM1/IMQ. (**I**) mAb-DXd/IMQ. (**J**) mAb-MMAF/IMQ.
