# Supplementary material for: Advanced CD276-Targeting Dual-Payload Antibody–Drug Conjugates for Cancer Therapy
Source: Cancer Res Commun. 2026 Apr 21;6(4):898–912. doi: 10.1158/2767-9764.CRC-26-0059 (PMC13099120; doi:10.1158/2767-9764.CRC-26-0059)
Supplement: Figure S6 — shows in vitro cytotoxicity evaluation at low drug concentrations. [file crc-26-0059_figure_s6_suppsf6.docx]

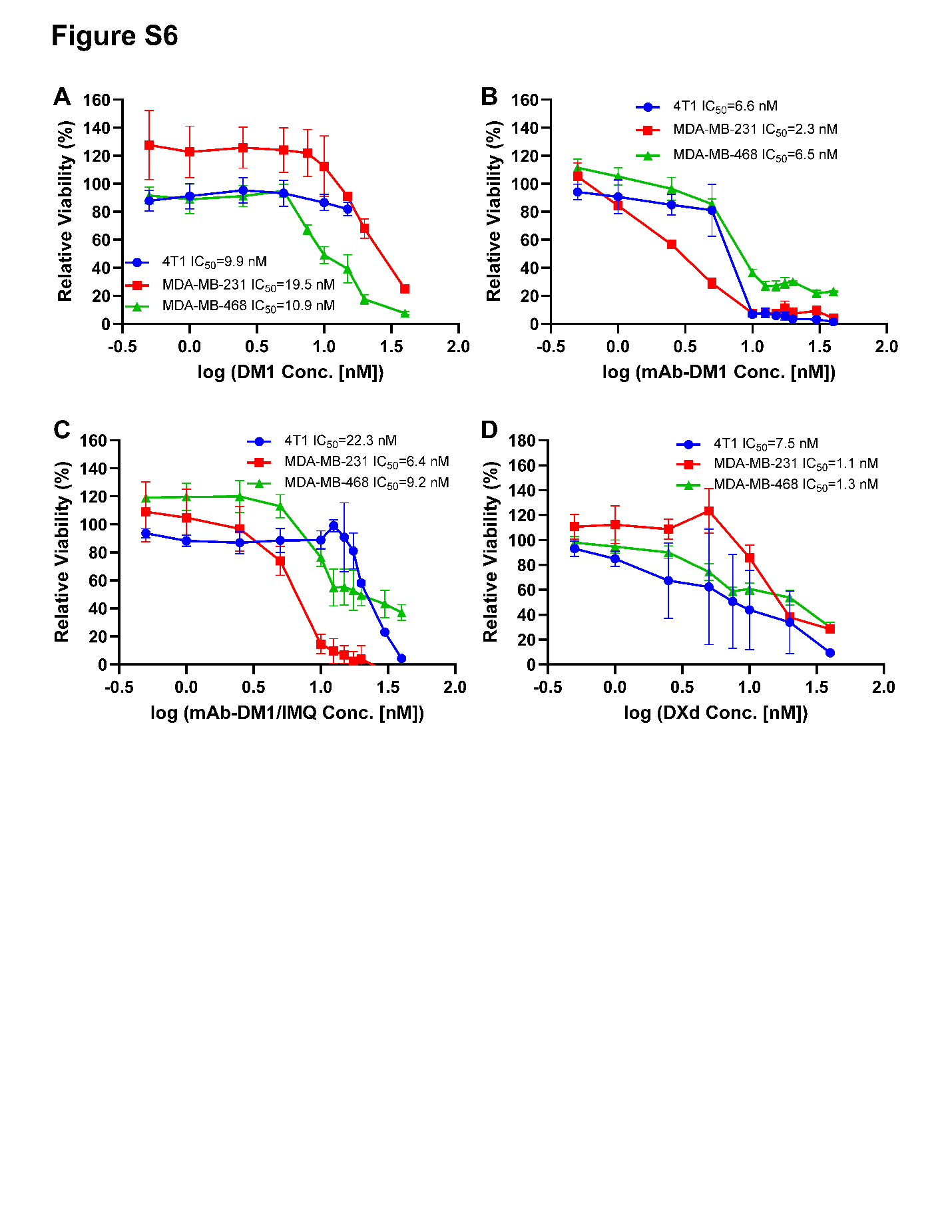


**Figure S6. *In vitro* cytotoxicity evaluation at low drug concentrations.** (**A-C**) Cytotoxicity of free DM1, mAb-DM1 ADC, and mAb-DM1/IMQ in TNBC cells (n=3). (**D**) Cytotoxicity of free DXd in TNBC cells (n=3).
