# Supplementary material for: Advanced CD276-Targeting Dual-Payload Antibody–Drug Conjugates for Cancer Therapy
Source: Cancer Res Commun. 2026 Apr 21;6(4):898–912. doi: 10.1158/2767-9764.CRC-26-0059 (PMC13099120; doi:10.1158/2767-9764.CRC-26-0059)
Supplement: Figure S7 — shows bulk RNA-Seq analysis of immune functions in TME. [file crc-26-0059_figure_s7_suppsf7.docx]

**
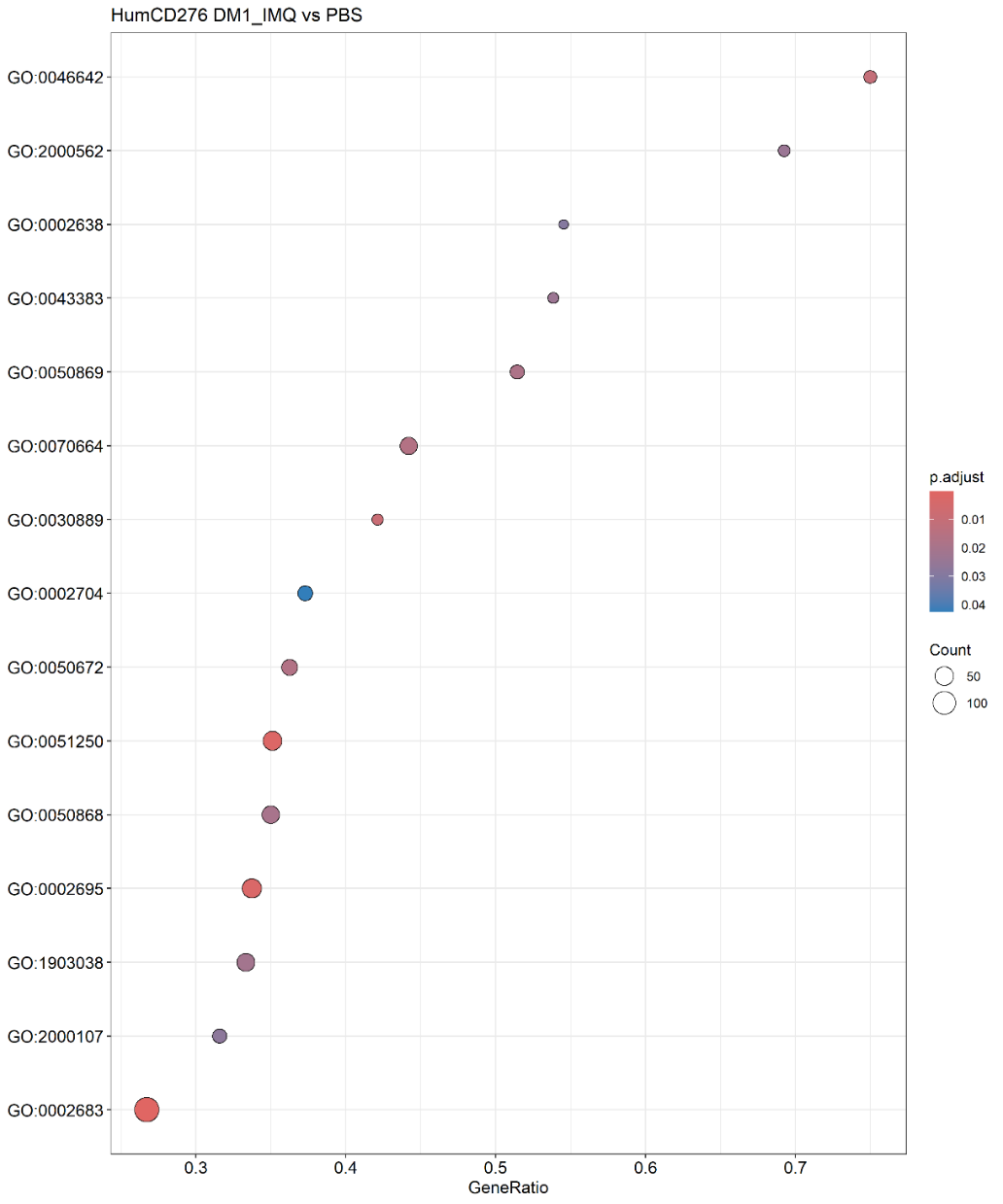
**

**Figure S7. Bulk RNA-Seq analysis of immune upregulation in TME.** The comparison was performed between mAb-DXd/IMQ group and saline group. n=4/group. GO 0046642: regulation of alpha-beta T cell proliferation; GO 2000562: regulation of CD4-positive, alpha-beta T cell proliferation; GO 0002683: regulation of immune system process; GO 0043383: T cell selection; GO 0050869: regulation of B cell activation; GO 0070664: regulation of leukocyte proliferation; GO 0030889: regulation of B cell proliferation; GO 0002704: regulation of leukocyte mediated immunity; GO 0050672: regulation of lymphocyte proliferation; GO 0051250: regulation of lymphocyte activation; GO 0050868: regulation of T cell activation; GO 0002695: regulation of leukocyte activation; GO 1903038: regulation of leukocyte cell-cell adhesion; GO 2000107: regulation of leukocyte apoptotic process; GO 0002638: regulation of immunoglobulin production.
